# Foreign Body in the Ear

**Published:** 1879-01-20

**Authors:** W. D. Hunt

**Affiliations:** Ky.


### *FOREIGN BODY IN THE EAR.*

BY W. D. HUNT, M. D., OF KY.

A negro boy came to me recently, complaining of very severe pain in his left ear and head, stating that he accidentally got something in his ear while thrashing garden peas about two years since, but thought he got it out, whatever it was. I had him lay down in a good light on his right side; having introduced the ear speculum, I discovered a foreign white-looking substance pressing against the membrana tympani, and nearly filling the cavity of the tympanum. I made an effort to extract it with dissecting forceps, but for the amount of tenderness and severe pain, I was compelled to desist. Said he, "I can't stand to have it taken out." Well, said I, die with it in there. I then proposed to give him cholroform and extract it without pain, but he objected, stating that it "killed folks—wont take it;" but he finally concluded to take it. I laid him down as above mentioned; having anæsthetized him, I introduced the speculum, and extracted a large May pea, very much enlarged, and partially decayed, giving almost instant relief.

The report of this case is on account of the great length of time the pea had remained in the ear without having germinated, or given rise to pain earlier than it did.

